# Virulence evolution during a naturally occurring parasite outbreak

**DOI:** 10.1007/s10682-022-10169-6

**Published:** 2022-04-12

**Authors:** Camden D. Gowler, Haley Essington, Bruce O’Brien, Clara L. Shaw, Rebecca W. Bilich, Patrick A. Clay, Meghan A. Duffy

**Affiliations:** 1 Department of Ecology and Evolutionary Biology, University of Michigan, 48104 Ann Arbor, MI USA; 2 Department of Biology, The Pennsylvania State University, University Park, 16802 Pennsylvania, PA USA

**Keywords:** *Pasteuria ramosa*, *Daphnia dentifera*, Eco-evolution, Virulence, Resistance, Infectivity

## Abstract

Virulence, the degree to which a pathogen harms its host, is an important but poorly understood aspect of host-pathogen interactions. Virulence is not static, instead depending on ecological context and potentially evolving rapidly. For instance, at the start of an epidemic, when susceptible hosts are plentiful, pathogens may evolve increased virulence if this maximizes their intrinsic growth rate. However, if host density declines during an epidemic, theory predicts evolution of reduced virulence. Although well-studied theoretically, there is still little empirical evidence for virulence evolution in epidemics, especially in natural settings with native host and pathogen species. Here, we used a combination of field observations and lab assays in the *Daphnia*-*Pasteuria* model system to look for evidence of virulence evolution in nature. We monitored a large, naturally occurring outbreak of *Pasteuria ramosa* in *Daphnia dentifera*, where infection prevalence peaked at ~ 40% of the population infected and host density declined precipitously during the outbreak. In controlled infections in the lab, lifespan and reproduction of infected hosts was lower than that of unexposed control hosts and of hosts that were exposed but not infected. We did not detect any significant changes in host resistance or parasite infectivity, nor did we find evidence for shifts in parasite virulence (quantified by host lifespan and number of clutches produced by hosts). However, over the epidemic, the parasite evolved to produce significantly fewer spores in infected hosts. While this finding was unexpected, it might reflect previously quantified tradeoffs: parasites in high mortality (e.g., high predation) environments shift from vegetative growth to spore production sooner in infections, reducing spore yield. Future studies that track evolution of parasite spore yield in more populations, and that link those changes with genetic changes and with predation rates, will yield better insight into the drivers of parasite evolution in the wild.

**Supplementary Information:**

The online version contains supplementary material available at 10.1007/s10682-022-10169-6.

## Introduction

By definition, parasites harm their hosts, but not all parasites harm their hosts to the same degree. Instead, the degree to which a parasite harms its host, known as virulence, varies depending on host and pathogen genotypes as well as the environment (Read 1994; Cressler et al. 2016). Moreover, virulence is not fixed but, rather, can evolve over time, with the evolutionary path depending on the ecological context (Galvani 2003). Because parasitism is common, important to the ecology and evolution of hosts, and of public health importance, there is extensive theory regarding the evolution of virulence in host-parasite interactions (Alizon et al. 2009; Cressler et al. 2016). However, empirical studies of the evolution of parasite virulence are much less common (Cressler et al. 2016). Therefore, we currently have a limited understanding of how parasites evolve, particularly during naturally occurring epidemics — and, as a result, are unable to predict how the amount of harm inflicted by parasites will change over time.

Early reasoning on the evolution of parasite virulence often concluded that parasites should evolve reduced virulence, to the point of no longer being parasites (Ewald 1983). However, this reasoning relies on group selection rather than individual selection (Lenski and May 1994) and, moreover, ignores the potential for tradeoffs between virulence and other key traits such as transmission (Frank 1996; Alizon et al. 2009; Cressler et al. 2016). Virulence evolution theory has become much more nuanced over the past few decades (e.g., Janoušková and Berec 2020; Pandey et al. 2022), and it is now recognized that parasites can evolve increased or decreased virulence, or, indeed, display no detectable change in virulence (Bolker et al. 2010; Cressler et al. 2016; Raymond and Erdos 2022; Visher et al. 2021).

In general, theory predicts that natural selection can favor a parasite maximizing the net population growth rate, *r*, or, alternatively, maximizing the number of secondary infections in the absence of competition, *R*_*0*_ (Frank 1996; Bolker et al. 2010). Which of these is favored depends on the ecological dynamics. At the beginning of an epidemic, selection should favor higher *r* — that is, selection should favor growing quickly (which is often associated with high virulence) even if that yields fewer secondary infections (Frank 1996). To understand this, it helps to consider two parasites: one that grows slowly within a host, causing a relatively small number of secondary infections per day (e.g., 1 per day) for a relatively long time (e.g., 10 days) vs. one that grows rapidly within a host, causing more secondary infections per day (e.g., 2 per day) but killing the host rapidly (e.g., within 2 days). During an epidemic, the latter parasite (which is more virulent based on the effect on host mortality) would be favored because it has a higher *r*, even though it produces fewer secondary infections (Frank 1996). As the epidemic progresses and there are fewer susceptible hosts to infect, evolution begins to favor lifetime reproductive fitness over rapid growth (Frank 1996; Bolker et al. 2010; Berngruber et al. 2013) — that is, to favor the slower growing, less virulent parasite in the example given above. Lower virulence is especially favored during epidemics that reduce host density (Lenski and May 1994; Frank 1996). When a disease is at equilibrium (that is, has become endemic), evolution will select for higher *R*_*0*_ (Frank 1996). Putting these pieces together, theory suggests that virulence should be higher during epidemics (as compared to endemic dynamics) and that optimal virulence should be particularly high early in an epidemic (Frank 1996; Bolker et al. 2010); moreover, if initial prevalences are low during an epidemic, that should lead to particularly large increases in virulence during the epidemic (Berngruber et al. 2013).

Some empirical studies have tracked virulence evolution on ecological time scales, both in the lab (e.g., Boots and Mealor 2007; Berngruber et al. 2013; White et al. 2020) and in nature. Perhaps the best-known study of virulence evolution occurred due to the introduction of myxoma virus to control invasive rabbit populations in Australia (Fenner and Ratcliffe 1965). In that system, very virulent strains of the virus predominated at the start, but pathogen and host evolution led to less virulent strains taking over (Dwyer, Levin, and Buttel 1990). However, more recently, the virus has evolved to once again be highly lethal, this time as a result of inducing immune collapse in its hosts (Kerr et al. 2017). In another well-studied system — the mycobacterium (*Mycoplasma gallisepticum*) that infects house finches in the United States — both host resistance and pathogen virulence changed over time (Hawley et al. 2013; Fleming-Davies et al. 2018; Gates et al. 2021); as predicted theoretically, virulence increased rapidly as the bacterium emerged (Hawley et al. 2013). In both of these systems, the hosts and/or pathogens were introduced by humans; this is notable because it means the best examples of virulence evolution in the wild come from systems where the host and parasite do not share a long coevolutionary history.

Other studies have demonstrated the value of using tractable model systems to study parasite evolution. For example, two recent experiments with *C. elegans* demonstrated that the evolution of virulence and infectivity of bacterial parasites was influenced by host diversity (White et al. 2020; Ekroth et al. 2021) — though, interestingly, those two studies found opposite effects, with one finding genetically diverse hosts drove higher parasite virulence (Ekroth et al. 2021) and the other finding lower virulence in genetically diverse hosts (White et al. 2020). Another system that has been used to understand evolution in host-parasite interactions is the *Daphnia* (zooplankton host)-*Pasteuria ramosa* (bacterial parasite) system (Ebert 2008; Ebert et al. 2016; Wale and Duffy 2021). One advantage of this system is that it allows for studies that bridge between the field and the lab (McLean and Duffy 2020); another is that hosts and parasites both produce long-lived resting stages that are incorporated into sediments (Decaestecker et al. 2004). A study that took advantage of this latter feature found that *P. ramosa* evolved differences in its within-host growth rate and effects on fecundity (Decaestecker et al. 2007); because that study used hosts and parasites from sediment cores, it looked at longer term dynamics of host-parasite coevolution over a span of several decades and combined hosts and parasites produced over multiple years. A different study carried out on a shorter time scale found that *P. ramosa* evolved to grow more rapidly while the epidemic progressed, although alternative explanations (especially phenotypic plasticity) were not ruled out (Auld et al. 2014b). More recently, a study of field-collected individuals found that the resource environment influences virulence in this system (measured as relative fecundity of infected hosts), with virulence being lower under the low food conditions that are typical of natural populations (Savola and Ebert 2019).

Here, we combine studies of ecological and evolutionary dynamics of *Daphnia dentifera* and *P. ramosa* during a naturally occurring disease outbreak. We tracked the prevalence of infection in a natural lake population. At three points during the epidemic, we collected hosts and parasites from the population, establishing them in the lab. We then used these to assess whether parasite infectivity, host resistance, parasite virulence and/or parasite spore yield evolved over the course of the epidemic.

## Materials and methods

### Study system

We focused on a host species, *Daphnia dentifera*, that is dominant in stratified lakes in the Midwestern United States (Tessier and Woodruff 2002). One of the most common pathogens in our study populations is the endospore-forming bacterium, *Pasteuria ramosa* (Gowler et al. 2021). *P. ramosa* infects its host through the gut and replicates itself within the hemolymph (Ebert et al. 2016). This pathogen exhibits strong genotype by genotype interactions with the host (Carius et al. 2001; Duneau et al. 2011) and is capable of evolution over relatively short periods of time (Decaestecker et al. 2007; Auld et al. 2014a). *P. ramosa* spores are long lasting (Decaestecker et al. 2004, 2007) and can be stored in the lab to infect hosts at a later date (Duffy and Hunsberger 2018).

*P. ramosa* is an obligate killing, sterilizing parasite (Ebert 2008). *P. ramosa* has the strongest impact on host fitness via its effects on reproduction (Ebert 2008; Auld et al. 2012; Clerc et al. 2015), particularly by reducing the number of clutches a host produces (and, therefore, impacting lifetime reproduction). *P. ramosa*’s fitness is indirectly impacted by host reproduction; as a sterilizing pathogen, it shunts host resource allocation away from reproduction (and often towards host growth), which increases the resources available to the parasite within the host (Ebert 2008; Cressler et al. 2014).

There are several reasons to expect *P. ramosa* might exhibit transient virulence evolution (Bolker et al. 2010). First, it harbors abundant genetic variation (Carius et al. 2001; Mouton and Ebert 2008; Luijckx et al. 2011). Second, because *P. ramosa* is a parasitic castrator, parasites that develop relatively slowly in a host can ultimately produce more spores (Jensen et al. 2006), but only if external sources of mortality do not kill the host first (Auld et al. 2014a); this means that the optimal rate of growth of the parasite within the host likely depends strongly on external sources of mortality, especially predation. Third, *P. ramosa* shows periodic outbreaks in natural populations (e.g., Duncan and Little 2007; Auld et al. 2014b; Gowler et al. 2021), meaning the epidemiological context (and, therefore, parasite traits that are favored) shifts over time.

### Epidemic dynamics

We monitored infection dynamics in lakes in Southeastern Michigan in July through November 2017. We initially studied two populations, but this became limited to one (Little Appleton Lake, Southeast Michigan, US) because we were unable to carry out the lab assays for the second lake due to lab access limitations during the COVID-19 pandemic.

Little Appleton Lake was sampled between 27 July and 13 November 2017 (11 samples total; mean interval between sampling dates = 11 days, median = 12 days). On each sampling day, we collected three replicate samples from the lake. Each of these samples contained three whole-water-column tows taken with a Wisconsin net, and each of the three tows was from a different sampling location in the deep basin of the lake; prior work has indicated that there is not significant spatial variation in the distribution of infected adults (Hall et al. 2005; E. Davenport and M.A. Duffy, unpubl. data). One of these samples was analyzed live within 24 h of collection to determine whether hosts were infected; hosts were visually diagnosed for pathogen infections under a dissection microscope (for late-stage infections) or compound microscope (for earlier infections). We analyzed infections in at least 200 *D. dentifera* (subsampled randomly) or, if there were fewer than 200 *D. dentifera* in the sample, for the whole sample. The other two samples were preserved in 90% ethanol; one of these preserved samples was later counted to determine *D. dentifera* density (as in Gowler et al. 2021). We analyzed infections in all *D. dentifera* (including juvenile females, adult asexual females, adult sexual females, and males) in the subsample; infections were seen in adult asexual females and juvenile females; no sexual females were observed to be infected with *P. ramosa*, and we found only 1 *P. ramosa*-infected male (out of 74 males analyzed from this lake during this study).

### Collection of host isofemale lines and parasite isolates

We collected *D. dentifera* that were infected with *P. ramosa* so that we could experimentally quantify parasite virulence and host resistance at different time points of the epidemic. We began collecting infected hosts once *P. ramosa* infections in the *D. dentifera* population reached a prevalence > 2%; we collected infected hosts roughly once every three weeks until we could no longer collect them because the prevalence was too low. At each of these collection points, we collected up to 30 infected hosts and kept them alive in the lab. We cured them of their bacterial infections with 0.025 g/mL of tetracycline, and maintained each one individually as clonal lines once they were uninfected. To control for epigenetic and environmental influences, we used maternal lines (Plaistow et al. 2015), taking third or later clutch hosts for at least three generations. *D. dentifera* clones were maintained at 16:8 light:dark and 20 ℃ in 30mL filtered lake water, and were fed 1,000,000 cells *Ankistrodesmus falcatus* (a nutritious green algae) four times per week.

Additionally, to collect pathogen samples from various points in the epidemic, we collected and froze up to 50 infected hosts per time point and preserved them in the freezer. We re-cultured pathogen spores by infecting *Daphnia* with spores collected from the same time point (e.g., pooled spores from time point 2 were cultured using a mixture of *D. dentifera* clones from time point 2) under standard conditions. We inoculated individual neonate (< 24 h old) *D. dentifera* from a mixture of genotypes from a given time period with 5,000 spores per mL in 2mL well plates for 48 h. After exposure, hosts were transferred to 100mL beakers of spore-free, filtered lake water in groups of five, and collected after host death to serve as parasite stock for the infection assays. We re-cultured the parasite because pathogen traits can be influenced by host genotype, temperature, and time spent in storage (Searle et al. 2015; Duffy and Hunsberger 2018; Shocket et al. 2018); rearing them under standard conditions minimizes variation due to plasticity. However, it is possible (perhaps even likely) that this process influenced the genetic composition of the *Pasteuria* spores that we used as our parasite stock for the infection assays.

### Infection assays

We inoculated individual neonate (< 24 h old) *D. dentifera* with the propagated *P. ramosa* spores from a given time point in well plates (one neonate per 2 mL well, 5,000 spores per mL, 48 h of exposure at 20 ℃). We reared the *D. dentifera* in conditions that yielded female offspring for this experiment; our sampling of the population (see “Epidemic dynamics” section above) found that ~ 3% of the individuals that we collected during this study were males, and only one of these was infected. Therefore, assessing the virulence of the parasite on females is most relevant to the conditions in the field during this study, though we note that, in a different *Daphnia* species, within-host dynamics of *P. ramosa* differ between male and female hosts, with consequences for pathogen evolution (Hall and Mideo 2018).

We used hosts and parasites collected on 28 August, 18 September, and 7 October 2017; hereafter, these are referred to as time points 1, 2, and 3, respectively. For each time point, we aimed to expose 10 replicates of each *D. dentifera* clone to the propagated pathogen stock from the same time point; however, in some cases, fewer individuals were available. The number of *D. dentifera* clones in each time point ranged from 4 to 14. To study host evolution, additional hosts from time point 3 were exposed to pathogens from time point 1. Ideally, we would have also exposed hosts from time point 1 to parasites from time point 3, since this would have allowed us to glean more information about evolution over the epidemic; unfortunately, we did not have enough individuals from time point 1 host clones to carry out these exposures. We also randomly selected genotypes from each time point to use as controls (selecting from the genotypes that we exposed to parasites), aiming for 5 replicates per host clone; these allowed us to compare the lifespan and reproduction of unexposed hosts with those of infected hosts. Reproduction was quantified by counting the number of clutches produced by each individual. Table A1 contains details about the number of host clones from each time point and the number of replicates per clone x exposure combination. These exposures were done in February 2019. After exposure, hosts were maintained individually in 30mL of filtered lake water, fed in the same manner as the maternal lines, and checked daily for mortality. Upon host death, hosts were placed in 1.5 mL tubes with 100 µL nanopure water and stored at -20 °C. Hosts were then ground to release spores, and spores were counted using a hemocytometer.

We first used these data to assess the impact of infections on hosts. We did this by comparing host lifespan and the number of clutches produced per host for individuals that were unexposed controls, individuals that were exposed and infected, and individuals that were exposed but remained uninfected. Next, we analyzed whether parasite infectivity and/or host resistance evolved over the course of the epidemic (by comparing the proportion of hosts that became infected when exposed to parasites from the same time point — that is, for “contemporary exposures” — and also by comparing the proportion of time 3 hosts that became infected when exposed to parasites from time 1 vs. time 3). Finally, we analyzed whether parasite virulence (host lifespan, host reproduction) and/or parasite fitness proxies (spore yield from infected hosts, parasite growth rate within hosts) changed over time for contemporary exposures and/or when hosts from time 3 were exposed to parasites from time 1 vs. time 3.

### Statistical analysis

To assess the overall impact of infection on hosts, and to see if there is a fitness cost associated with resisting infection, we analyzed data on lifespan and reproduction of infected, exposed but uninfected, and unexposed control hosts. Specifically, we analyzed whether there was a difference in the lifespan (measured in days) of these three classes of hosts using mixed effects models with exposure class (infected, exposed but uninfected, and unexposed controls) as a fixed effect and host clone as a random effect. Because of overdispersion, we used negative binomial generalized linear mixed effects models (GLMMs), using glmer.nb from the lme4 package in R (Bates et al. 2015). We used the emmeans package (Lenth 2021) to estimate pairwise contrasts between these three groups. We used the same model structure and approach to analyze data on reproduction, but this time using the number of clutches produced per host individual as the response variable; these were the same host individuals that were used in the lifespan analysis.

We next analyzed data on infection prevalence in our infection assays. These data are binomially distributed, so these models were run with a binomial error distribution. Because of overdispersion in the data, we included host clone as a random effect in the model. Therefore, we analyzed data on whether hosts became infected or not using mixed effects logistic regression (using glmer from the lme4 package in R; Bates et al. 2015) with parasite time point as a fixed effect factor and host clone as a random effect. We had two models: one with infection outcomes (infected or uninfected) for hosts from each of the three time points when exposed to parasites from the same time point, and a second with infection outcomes for hosts from time point 3 when exposed to parasites from time 1 or time 3.

Finally, we analyzed data on parasite virulence (lifespan and reproduction of infected hosts) and parasite fitness proxies (spore yield from infected hosts, parasite growth rate within hosts). As with the infectivity data, we did this both for host clones exposed to parasites from the same time point (“contemporary” pairings) and for hosts from time point 3 that were exposed to parasites from time 1 or time 3. We looked for a change in impacts of parasites on host lifespan by analyzing data on the lifespan for infected hosts. Because of overdispersion, we used negative binomial generalized linear mixed effects models (GLMMs), using glmer.nb from the lme4 package in R (Bates et al. 2015). This model included parasite time point as a fixed effect and host clone as a random effect. We also used negative binomial glmms to analyze data on reproduction. More specifically, we analyzed the number of clutches produced per host individual; we did not have information on the number of offspring per clutch, but, given that *P. ramosa* primarily affects the number of clutches (because it is a sterilizing parasite) rather than the number of individuals per clutch, we expect the number of clutches to strongly correlate with lifetime fecundity. We analyzed log spore yield from infected hosts using a linear mixed effects model (with Gaussian error distribution), with parasite time point as a fixed effect and host clone as a random effect. Parasite growth rate was a composite metric that was calculated by dividing the total number of spores produced per infected host by the lifespan of that host individual. Parasite growth rate was also analyzed with a linear mixed effects model with Gaussian error distribution, parasite time point as a fixed effect, and host clone as a random effect. For all four of these metrics (lifespan, number of clutches, log spore yield, parasite growth rate), we ran the analysis once for contemporary pairings and once for time 3 hosts exposed to time 1 vs. time 3 parasites. All analyses were performed using R version 4.1.2 (R Core Team 2021), with data manipulation and visualization using the tidyverse (Wickham et al. 2019) and cowplot (Wilke 2020) packages.

## Results

### Epidemic dynamics

**Fig. 1:**
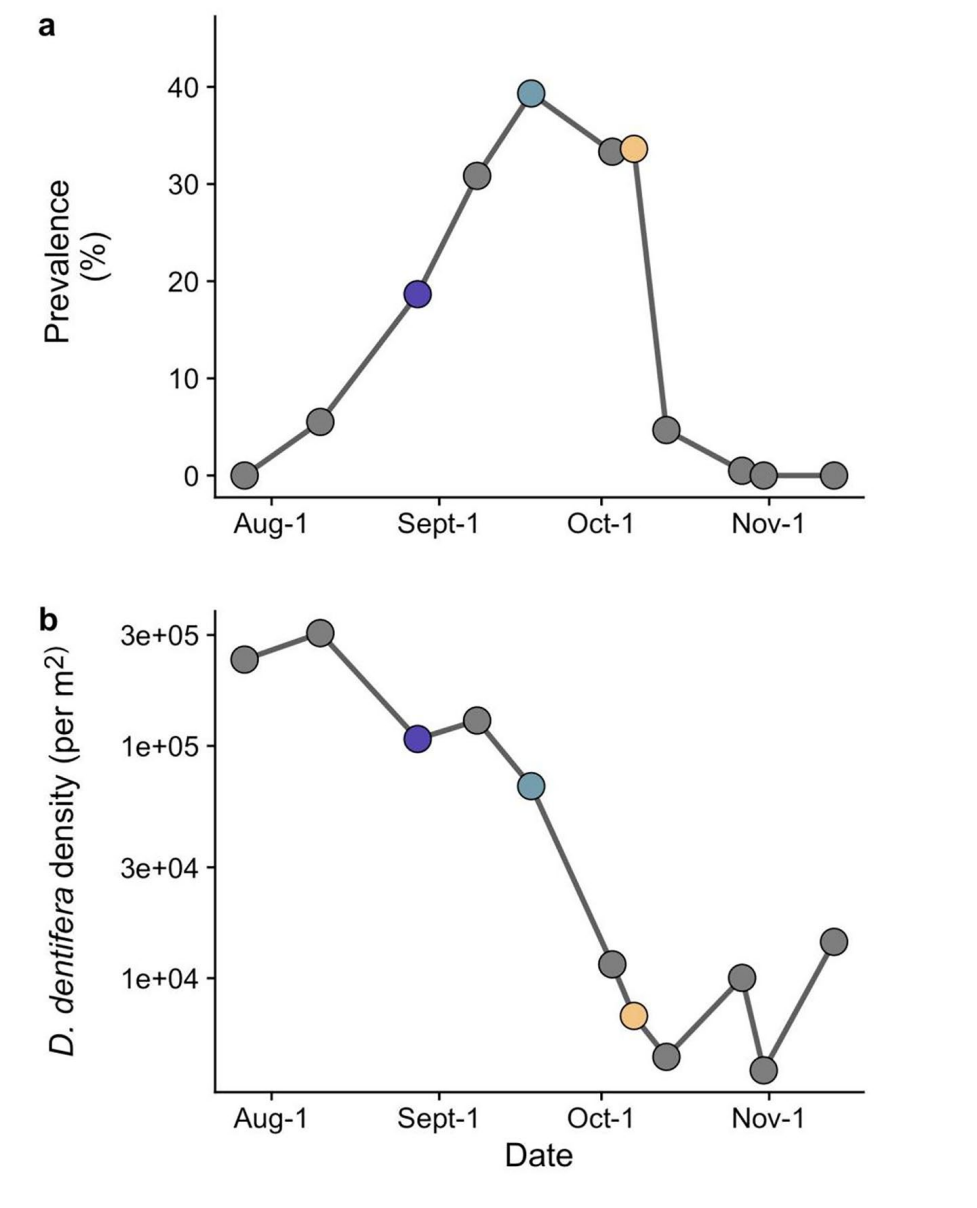
*Daphnia dentifera* in Little Appleton Lake experienced a large epidemic of *Pasteuria ramosa;* host density decreased substantially during the epidemic. (a) Prevalence of *P. ramosa* increased steadily from the beginning of sampling, peaked at 39% of hosts infected, and decreased more sharply during October. (b) *D. dentifera* density was high at the beginning of August and decreased during September and the first part of October. Host and parasite samples were collected at three time points throughout the epidemic trajectory in the Fall of 2017; these three timepoints are indicated with colors that match the timepoints in Figs. 3 and 4

There was a large epidemic of *P. ramosa* in *D. dentifera* in this population. At the peak, the percentage of infected *D. dentifera* reached nearly 40% of the total population (Fig. 1a, time 2), which is a large outbreak for Midwestern lake populations. *D. dentifera* density declined throughout the epidemic (Fig. 1b).

### Impacts of infection on hosts

**Fig. 2:**
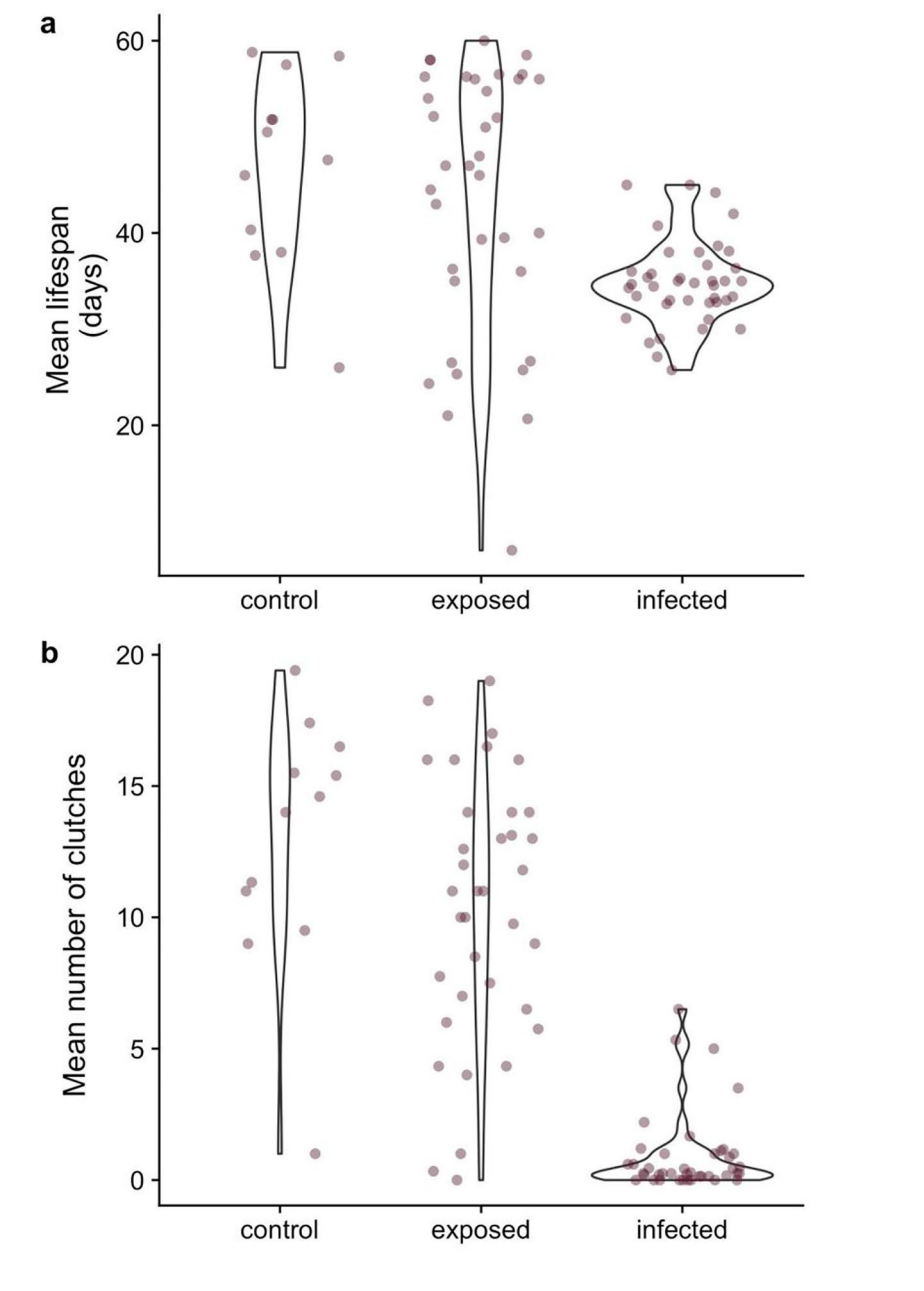
*D. dentifera* that were infected with *P. ramosa* had shorter lives and many fewer clutches than unexposed control hosts; there was no significant difference between the lifespan and reproduction of control hosts and hosts that were exposed but not infected. Statistical analyses used individual-level data; in order to more clearly visualize the data, averages for each host clone x parasite exposure combination are plotted

In laboratory infection assays, infected hosts died sooner than unexposed control hosts and hosts that were exposed but uninfected (Fig. 2a; contrasts: control vs. infected: *Z* = 6.7, p < 0.0001, exposed but uninfected vs. infected: *Z* = 6.3, p < 0.0001, control vs. exposed but uninfected: *Z* = 1.8, p = 0.17). Infected hosts produced many fewer clutches than unexposed control hosts and hosts that were exposed but uninfected (Fig. 2b; contrasts: control vs. infected: *Z* = 14.4, p < 0.0001, exposed but uninfected vs. infected: *Z* = 17.3, p < 0.0001, control vs. exposed but uninfected: *Z* = 0.73, p = 0.74). These analyses combined host clones from different time points; lifespan and reproduction of infected hosts at different time points are presented below.

### Changes in infectivity and/or resistance

**Fig. 3:**
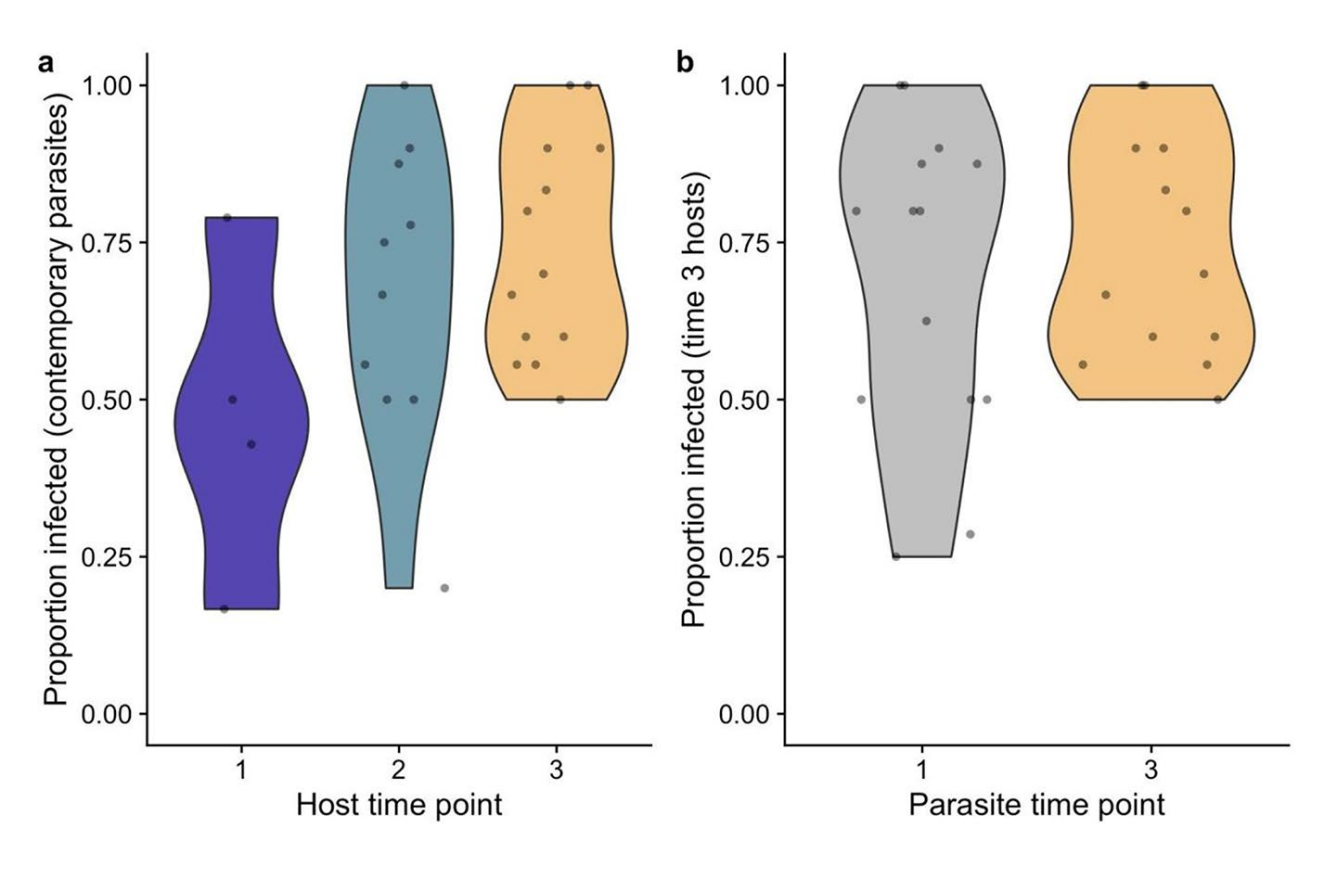
There was no difference in the proportion of hosts that became infected when hosts from a given time point were exposed to contemporary parasites (panel a), nor when time 3 hosts were exposed to parasites from time 1 vs. time 3 (panel b)

When hosts were exposed to parasites from the same time point in the laboratory, there was no difference in the proportion that became infected over time (Fig. 3a; χ^2^ = 2.58, p = 0.28). When looking just at hosts from time point 3, there was no difference in the proportion infected when these hosts were exposed to parasites from time 1 vs. time 3 (Fig. 3b; χ^2^ = 0.72, p = 0.40).

### Changes in parasite virulence and fitness

**Fig. 4:**
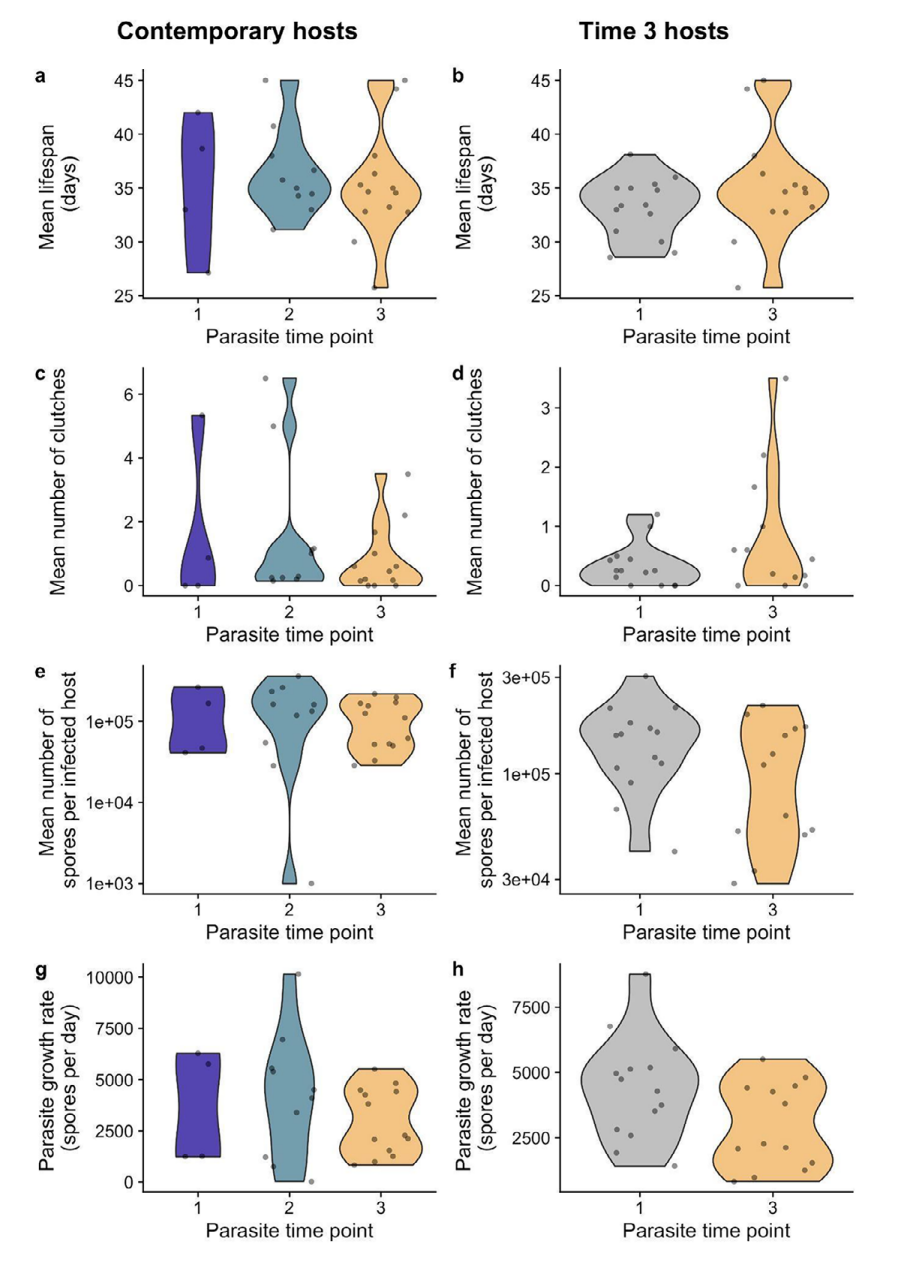
Virulence of parasites against contemporary host clones did not significantly differ across the three time points, nor did the impact of parasites from two different time points on time 3 hosts; however, time 3 parasites yielded fewer spores and had a slower within host growth rate in time 3 hosts, as compared to time 1 parasites. Left panels: virulence of parasites against hosts from the same time point (e.g., when hosts from time 2 were exposed to parasites from time 2). Right panels: virulence of parasites from time 1 and time 3 in hosts from time 3; this allows for isolation of the effects of parasite evolution. There were no significant differences in lifespan (a&b) or reproduction (c&d). The number of spores produced per infected host, and the parasite growth rate within infected hosts, did not differ significantly for hosts from the three time points exposed to their contemporary parasites (e&g). However, when time 3 hosts were exposed to parasites from time 1 vs. time 3, hosts infected with time 1 parasites produced significantly more spores (f) and had a significantly faster growth rate (h); this suggests that the parasite evolved to grow slower and produce fewer spores, which was contrary to our expectations. Statistical analyses used individual-level data; in order to more clearly visualize the data, averages for each host clone x parasite exposure combination are plotted

There was no difference in lifespan or reproduction of hosts when exposed to contemporary parasites (Fig. 4a&c; lifespan: χ^2^ = 2.93, p = 0.23; reproduction: χ^2^ = 1.96, p = 0.38), nor was there a difference in lifespan or reproduction of time 3 hosts when exposed to spores from time 1 vs. time 3 (Fig. 4b&d; lifespan: *Z* = 1.25, p = 0.21; reproduction: *Z* = 1.12, p = 0.26). For hosts exposed to contemporary parasites, there was no significant difference in spore yield or parasite growth rate across the three time points (Fig. 4e&g; log spore yield: χ^2^ = 2.17, p = 0.34; parasite growth rate: χ^2^ = 3.65, p = 0.16).

There was a signature of parasite evolution in a direction opposite of what was expected: time 3 hosts that were exposed to time 3 parasites produced significantly fewer spores than time 3 hosts exposed to time 1 parasites (χ^2^ = 10.4, p = 0.0012; Fig. 4f). This was associated with a significantly lower growth rate for parasites from time 3 (χ^2^ = 8.96, p = 0.0028; Fig. 4 h).

## Discussion

There was a large outbreak of a highly virulent parasite in this population, and population size decreased substantially during this outbreak. Despite this, there was no significant change in the virulence or growth rate of parasites when they infected hosts from the same time point, nor was there a change in the virulence of the parasite when measured in terms of host lifespan or the number of clutches produced. However, between the first and third time points at which hosts and parasites were collected, the parasite evolved to produce significantly fewer spores in infected hosts and to have a slower growth rate.

Based on theory related to transient virulence evolution (Frank 1996; Bolker et al. 2010), we expected virulence to be highest at the start of the epidemic, and for virulence to decrease as parasite prevalence increased. We expected this because maximizing the intrinsic growth rate (*r*) is thought to be the optimal strategy for the parasite at the early stages of epidemics (Frank 1996; Bolker et al. 2010), and also because there was a strong decrease in host population density during the epidemic, which should also favor lower virulence (Lenski and May 1994; Frank 1996). Therefore, we expected relatively strong impacts of parasites from early in the epidemic on host lifespan and/or the number of clutches produced by infected hosts, and for those effects to decrease by later in the epidemic. However, our results do not match this pattern — we did not find a significant change in virulence in terms of host lifespan or the number of clutches produced. Moreover, while parasite growth rate decreased over the epidemic, as predicted by virulence evolution theory, mean number of spores per infected host (a proxy for *R*_*0*_) decreased as well, contrary to predictions.

Why did we not see a shift in parasite virulence over the course of the epidemic? The shift in spore yield from infected hosts (which was surprising on its own, as discussed more below) suggests that there was sufficient time for the parasite to evolve, and prior studies have found significant host evolution over similar time periods (e.g., Duffy et al. 2008; Duffy et al. 2012; Paplauskas et al. 2021). One possibility is that the parasite population evolved very rapidly at the beginning of the epidemic, prior to us isolating hosts and parasites (Fig. 1a). The lifespan of infected hosts is ~ 35 days (Fig. 2a), and it takes at least 14 days for the parasite to develop transmission stages at 20 ℃. Factoring in higher predation on infected hosts in the field (Duffy et al. 2019), which most likely results from the increased opacity of infected hosts (Wale et al. 2022), this suggests a maximum of ~ 2–3 rounds of transmission of this obligate killer between when the parasite was first detected and when we isolated hosts and parasites. While this could be sufficient time for the parasite to evolve, we would have expected that selection on the parasite would still be strong between our first time point and our third, including because there was a very strong decrease in host density across this time (Fig. 1b). A second possibility is that the spores from infected hosts isolated at the second and third time points may have originated (much) earlier. Some spores may have been generated early in the epidemic and remained in the water column, whereas others may have been resuspended from a sediment spore bank, where spores can remain dormant for decades (Decaestecker et al. 2004, 2007). Because such time lags have the potential to strongly influence evolutionary dynamics, it would be extremely helpful to know more about the dynamics of spores in the water column (e.g., how frequently they are resuspended from the sediment, how long they can persist in the water column). Tracking parasite genotypes, including those in the water column and those successfully infecting hosts, will help us determine the time scales that are most relevant to ecological and evolutionary dynamics in this system.

Why did the parasite evolve to produce *fewer* spores per infected host? This change was not apparent when looking at contemporary host-parasite pairings, but became clear when hosts from the third time point were exposed to parasites from time point 1 vs. time point 3. Initially, the lower spore yield of parasites from time 3 seems unexpected, since the number of spores produced per infected host is a key component of parasite fitness. It is possible that this is a maladaptive change, but we think that it is more likely that this is “apparent maladaptation” due to interrelated fitness components (Brady et al. 2019). More specifically, we propose that the reduced spore yield from infected hosts might result from tradeoffs associated with parasite growth. In an earlier study, *P. ramosa* lines that experienced high host mortality evolved to produce transmission spores earlier in infections, at a cost of reduced overall spore yield (Auld et al. 2014a). A likely mechanism underlying this is the need to shift from vegetative growth to producing transmission spores at some point during infection; higher host mortality rates should select for parasite genotypes that make that shift sooner. Because we allowed hosts in our infection assays to die from senescence or effects of the parasite, our assay reflects a low predation environment. Thus, if this Little Appleton population experienced high mortality rates during this epidemic (e.g., due to selective fish predation), the shift to lower spore yields might actually be adaptive, reflecting a shift to earlier parasite reproduction; future studies that quantify predation rates and that assay spore yield at different time points after infection would be valuable. Moreover, it is likely that there was rapid evolution of the host. Indeed, the lack of a change in spore yield in contemporary host-parasite pairings, combined with the shift in spore yield of the parasite in time 3 hosts, suggests that hosts also have evolved during this study. In particular, the combination of no change in parasite growth rate for contemporary pairings (Fig. 4 g) and decreased growth rate when parasites from time 1 vs. time 3 were grown in the same host genotypes (Fig. 4 h) argues for changes in both host and parasite, with the parasite evolving to grow more slowly and the host evolving decreased resistance (where, in this case, “resistance” refers to the ability of the parasite to grow within the host, rather than the likelihood of infection). In future work, it would be valuable to track phenotypic and genetic changes in the host and parasite; a recent study found that *Daphnia magna* evolved rapidly in response to *P. ramosa* outbreaks and identified two genomic regions driving resistance (Ameline et al. 2020). Future work on this is especially important since, due to impacts of the COVID-19 pandemic, we were only able to track evolution in a single population.

Taken together, we found that the parasite evolved to produce fewer spores in infected hosts, but that this was not associated with a change in virulence (quantified as impacts on host lifespan and number of clutches produced) — in time 3 hosts, parasites from time 3 produced fewer spores than parasites from time 1, but hosts lived the same amount of time and had the same number of clutches regardless of parasite timepoint. What does this mean for links between host and parasite fitness? Hosts are sterilized early in infection and generally remain castrated for the remainder of the infection (though it is possible for hosts to sometimes reproduce again late in infections; Clerc et al. 2015). If *P. ramosa* successfully manipulates host energy allocation, hosts should stop reproducing and become larger, increasing the amount of energy available for the parasite. Thus, host lower reproduction should be associated with greater parasite spore yield, if all else is equal. However, as discussed in the previous paragraph, there can be strong selection on the parasite associated with host mortality rate, which could mean that a parasite that exerts strong control on host reproduction yields few spores as a result of shifting from vegetative growth to spore production relatively quickly after infection, complicating the relationship between host reproduction and parasite spore yield. The relationship between spore yield and host lifespan is also likely to be messy. A study on *Daphnia magna* and *P. ramosa* found that hosts that lived an intermediate amount of time after infection yielded the most spores (Jensen et al. 2006). Overall, the main link between host and parasite fitness in this system comes from whether or not a host becomes infected: host fitness is greatly reduced, and parasite fitness greatly increased if the parasite successfully infects the host; relationships between parasite spore yield, host lifespan, and host reproduction are likely to be more variable.

Evolution of parasites over the course of an epidemic can have strong impacts on ecological dynamics of host-parasite interactions. However, we still have relatively few studies regarding parasite evolution in the wild, particularly from naturally occurring outbreaks. Our study found that a common bacterial parasite evolved to produce fewer spores over the course of an epidemic. Future studies that track evolution of spore yield in more populations, and that link those changes with genetic changes and with predation rates in the field, will help us better understand the drivers of parasite evolution in the wild.

## Electronic Supplementary Material

Below is the link to the electronic supplementary material.

Supplementary Material 1

## Data Availability

Data and code are available here: https://doi.org/10.5061/dryad.b8gtht7db.

## References

[CR1] Alizon S, Hurford A, Mideo N, Van Baalen M (2009). Virulence evolution and the trade-off hypothesis: history, current state of affairs and the future. J Evol Biol.

[CR2] Ameline C, Bourgeois Y, Vögtli F, Savola E, Andras J, Engelstädter J, Ebert D (2020) A two-locus system with strong epistasis underlies rapid parasite-mediated evolution of host resistance. Mol Biol Evol 10.1093/molbev/msaa311 PMC8042741 33258959

[CR3] Auld SKJR, Hall SR, Duffy MA (2012). Epidemiology of a Daphnia-multiparasite system and its implications for the Red Queen. PLoS ONE.

[CR4] Auld SKJR, Hall SR, Housley Ochs J, Sebastian M, Duffy MA (2014). Predators and patterns of within-host growth can mediate both among-host competition and evolution of transmission potential of parasites. Am Nat.

[CR5] Auld SKJR, Wilson PJ, Little TJ (2014b) Rapid change in parasite infection traits over the course of an epidemic in a wild host–parasite population. Oikos 123:232–238

[CR6] Bates D, Mächler M, Bolker B, Walker S (2015). Fitting Linear Mixed-Effects Models Using lme4. J Stat Softw.

[CR7] Berngruber TW, Froissart R, Choisy M, Gandon S (2013). Evolution of virulence in emerging epidemics. PLoS Pathog.

[CR8] Bolker BM, Nanda A, Shah D (2010). Transient virulence of emerging pathogens. J R Soc Interface.

[CR9] Boots M, Mealor M (2007). Local Interactions Select for Lower Pathogen Infectivity. Science.

[CR10] Brady SP (2019). Understanding Maladaptation by Uniting Ecological and Evolutionary Perspectives. Am Nat.

[CR11] Carius HJ, Little TJ, Ebert D (2001). Genetic variation in a host-parasite association: Potential for coevolution and frequency-dependent selection. Evolution.

[CR12] Clerc M, Ebert D, Hall MD (2015) Expression of parasite genetic variation changes over the course of infection: implications of within-host dynamics for the evolution of virulence. Proceedings of the Royal Society B 282(1804):20142820 10.1098/rspb.2014.2820 PMC4375866 25761710

[CR13] Cressler CE, McLeod DV, Rozins C, Van Den Hoogen J, Day T (2016). The adaptive evolution of virulence: a review of theoretical predictions and empirical tests. Parasitology.

[CR14] Cressler CE, Nelson WA, Day T, McCauley E (2014) Starvation reveals the cause of infection-induced castration and gigantism. Proceedings of the Royal Society B: Biological Sciences 281(1792):20141087 10.1098/rspb.2014.1087 PMC4150321 25143034

[CR15] Decaestecker E, Gaba S, Raeymaekers JAM, Stoks R, Van Kerckhoven L, Ebert D, De Meester L (2007). Host–parasite ‘Red Queen’ dynamics archived in pond sediment. Nature.

[CR16] Decaestecker E, Lefever C, De Meester L, Ebert D (2004). Haunted by the past: Evidence for dormant stage banks of microparasites and epibionts of Daphnia. Limnol Oceanogr.

[CR17] Duffy MA, Brassil CE, Hall SR, Tessier AJ, Cáceres CE, Conner JK (2008) Parasite-mediated disruptive selection in a natural Daphnia population. BMC Evol Biol 8:80 10.1186/1471-2148-8-80 PMC2276202 18328099

[CR18] Duffy MA, Cáceres CE, Hall SR (2019) Healthy herds or predator spreaders? Insights from the plankton into how predators suppress and spread disease. Wildlife Disease Ecology

[CR19] Duffy MA, Hunsberger KK (2018). Infectivity is influenced by parasite spore age and exposure to freezing: do shallow waters provide Daphnia a refuge from some parasites?. J Plankton Res.

[CR20] Duffy MA, Ochs JH, Penczykowski RM, Civitello DJ, Klausmeier CA, Hall SR (2012). Ecological context influences epidemic size and parasite-mediated selection. Science.

[CR21] Duncan AB, Little TJ (2007). Parasite-driven genetic change in a natural population of Daphnia. Evolution.

[CR22] Duneau D, Luijckx P, Ben-Ami F, Laforsch C, Ebert D (2011). Resolving the infection process reveals striking differences in the contribution of environment, genetics and phylogeny to host-parasite interactions. BMC Biol.

[CR23] Ebert D (2008). Host-parasite coevolution: Insights from the Daphnia-parasite model system. Curr Opin Microbiol.

[CR24] Ebert D, Duneau D, Hall MD, Luijckx P, Andras JP, Du Pasquier L, Ben-Ami F (2016) A Population Biology Perspective on the Stepwise Infection Process of the Bacterial Pathogen Pasteuria ramosa in Daphnia. In: Rollinson D, Stothard JR (eds) Advances in Parasitology, vol 91. Academic Press, pp 265–310. doi:10.1016/bs.apar.2015.10.001 10.1016/bs.apar.2015.10.001 27015951

[CR25] Ekroth AKE, Gerth M, Stevens EJ, Ford SA, King KC (2021). Host genotype and genetic diversity shape the evolution of a novel bacterial infection. ISME J.

[CR26] Ewald PW (1983) Host-Parasite Relations, Vectors, and the Evolution of Disease Severity. Annu Rev Ecol Syst 14:465–485

[CR27] Fenner F, Ratcliffe FN (1965). Myxomatosis.

[CR28] Fleming-Davies AE (2018). Incomplete host immunity favors the evolution of virulence in an emergent pathogen. Science.

[CR29] Frank SA (1996). Models of parasite virulence. Q Rev Biol.

[CR30] Galvani AP (2003). Epidemiology meets evolutionary ecology. Trends Ecol Evol.

[CR31] Gates DE, Staley M, Tardy L, Giraudeau M, Hill GE, McGraw KJ, Bonneaud C (2021). Levels of pathogen virulence and host resistance both shape the antibody response to an emerging bacterial disease. Sci Rep.

[CR32] Gowler CD, Rogalski MA, Shaw CL, Hunsberger KK, Duffy MA (2021) Density, parasitism, and sexual reproduction are strongly correlated in lake Daphnia populations. Ecol Evol 11:10446–10456 10.1002/ece3.7847 PMC8328469 34367587

[CR33] Hall MD, Mideo N (2018) Linking sex differences to the evolution of infectious disease life-histories. Philos Trans R Soc Lond B Biol Sci 373(1757):20170431 10.1098/rstb.2017.0431 PMC6125729 30150228

[CR34] Hall SR, Duffy MA, Tessier AJ, Cáceres CE (2005). Spatial heterogeneity of daphniid parasitism in lakes. Oecologia.

[CR35] Hawley DM, Osnas EE, Dobson AP, Hochachka WM, Ley DH, Dhondt AA (2013). Parallel patterns of increased virulence in a recently emerged wildlife pathogen. PLoS Biol.

[CR36] Janoušková E, Berec L (2020). Fecundity-Longevity Trade-Off, Vertical Transmission, and Evolution of Virulence in Sterilizing Pathogens. Am Nat.

[CR37] Jensen KH, Little T, Skorping A, Ebert D (2006). Empirical support for optimal virulence in a castrating parasite. PLoS Biol.

[CR38] Kerr PJ (2017). Next step in the ongoing arms race between myxoma virus and wild rabbits in Australia is a novel disease phenotype. Proc Natl Acad Sci U S A.

[CR39] Lenski RE, May RM (1994). The evolution of virulence in parasites and pathogens - reconciliation between 2 competing hypotheses. J Theor Biol.

[CR40] Lenth RV (2021) emmeans: Estimated Marginal Means, aka Least-Squares Means

[CR41] Luijckx P, Ben-Ami F, Mouton L, Du Pasquier L, Ebert D (2011). Cloning of the unculturable parasite Pasteuria ramosa and its Daphnia host reveals extreme genotype–genotype interactions. Ecol Lett.

[CR42] McLean KD, Duffy MA, Banzhaf W (2020). Ecological Context Influences Evolution in Host-Parasite Interactions: Insights from the Daphnia-Parasite Model System. Evolution in Action: Past, Present and Future: A Festschrift in Honor of Erik D. Goodman.

[CR43] Mouton L, Ebert D (2008). Variable-number-of-tandem-repeats analysis of genetic diversity in Pasteuria ramosa. Curr Microbiol.

[CR44] Pandey A, Mideo N, Platt TG (2022) Virulence Evolution of Pathogens That Can Grow in Reservoir Environments. Am Nat 199(1):141–158 10.1086/717177 34978966

[CR45] Paplauskas S, Brand J, Auld S (2021) Ecology directs host–parasite coevolutionary trajectories across Daphnia–microparasite populations. Nature Ecology & Evolution 10.1038/s41559-021-01390-7 33589801

[CR46] Plaistow SJ, Shirley C, Collin H, Cornell SJ, Harney ED (2015). Offspring Provisioning Explains Clone-Specific Maternal Age Effects on Life History and Life Span in the Water Flea, Daphnia pulex. Am Nat.

[CR47] R Core Team (2021) R: A Language and Environment for Statistical Computing

[CR48] Raymond B, Erdos Z (2022) Passage and the evolution of virulence in invertebrate pathogens: Fundamental and applied perspectives. J Invertebr Pathol 187:107692 10.1016/j.jip.2021.107692 34798134

[CR49] Read AF (1994). The evolution of virulence. Trends Microbiol.

[CR50] Savola E, Ebert D (2019). Assessment of parasite virulence in a natural population of a planktonic crustacean. BMC Ecol.

[CR51] Searle CL, Ochs JH, Cáceres CE, Chiang SL, Gerardo NM, Hall SR, Duffy MA (2015). Plasticity, not genetic variation, drives infection success of a fungal parasite. Parasitology.

[CR52] Shocket MS (2018). Warmer is sicker in a zooplankton-fungus system: a trait-driven approach points to higher transmission via host foraging. Am Nat.

[CR53] Tessier AJ, Woodruff P (2002). Cryptic trophic cascade along a gradient of lake size. Ecology.

[CR54] Visher E et al (2021) The three Ts of virulence evolution during zoonotic emergence. Proc Biol Sci 288(1956):20210900 10.1098/rspb.2021.0900 PMC8354747 34375554

[CR55] Wale N, Duffy MA (2021) The use and underuse of model systems in infectious disease ecology & evolutionary biology. Am Nat 10.1086/714595 34143716

[CR56] Wale N, Fuller RC, Johnsen S, Turrill ML, Duffy MA (2022) The visual ecology of selective predation. Are unhealthy hosts less stealthy hosts? Ecol Evol 10.1002/ece3.8464 PMC8717294 35003695

[CR57] White PS (2020). Host heterogeneity mitigates virulence evolution. Biol Lett.

[CR58] Wickham H (2019). Welcome to the tidyverse. J Open Source Softw.

[CR59] Wilke CO (2020) cowplot: Streamlined Plot Theme and Plot Annotations for ‘ggplot2’

