# Supplementary material for: Virulence evolution during a naturally occurring parasite outbreak
Source: Evol Ecol. 2022 Apr 12;37(1):113–29. doi: 10.1007/s10682-022-10169-6 (PMC9002213; doi:10.1007/s10682-022-10169-6)
Supplement: Supplementary file 1 — Supplementary Material 1 [file 10682_2022_10169_MOESM1_ESM.docx]

**Appendix (for online publication)**

**Table A1. Summary of experimental combinations of host clones and parasites from different time points.** This table shows host clones from three different time points (28 August, 18 September, and 7 October 2017). The “**Contemporary Parasite Exposure**” section shows the exposures of host clones to parasites from the same time point (e.g., hosts from time 1 to parasites from time 1, etc.). The “Exposed” column indicates the number of host individuals (one per beaker) that were exposed to the parasite spores. The “Infected” column shows how many of them became infected; virulence measures (lifespan and number of clutches) and parasite spore yield could only be calculated for these infected individuals. The “**Unexposed**” section shows how many “control” individuals there were; these individuals were treated in the same way as the exposed animals except they were not exposed to any parasite spores. The “**Exposure to Earlier Parasite**” section only applies to Time 3 hosts; these columns indicate the number of individuals from each clone that were exposed to and infected by parasites from Time Point 1.

| **Host Time Point** | **Host**  **Clone** | **Contemporary Parasite Exposure** | | **Unexposed** | **Exposure to Earlier Parasite** | |
| --- | --- | --- | --- | --- | --- | --- |
|  |  | **Exposed** | **Infected** | **Control** | **Exposed** | **Infected** |
| 1 | 17 | 6 | 1 | 3 | NA | NA |
| 1 | 22 | 7 | 3 | 3 | NA | NA |
| 1 | 29 | 19 | 15 | 5 | NA | NA |
| 1 | 31 | 2 | 1 | 1 | NA | NA |
| 2 | 211 | 6 | 4 | 0 | NA | NA |
| 2 | 220 | 10 | 9 | 5 | NA | NA |
| 2 | 223 | 8 | 7 | 4 | NA | NA |
| 2 | 224 | 1 | 1 | 0 | NA | NA |
| 2 | 227 | 8 | 6 | 0 | NA | NA |
| 2 | 234 | 9 | 7 | 0 | NA | NA |
| 2 | 240 | 9 | 5 | 5 | NA | NA |
| 2 | 241 | 10 | 2 | 0 | NA | NA |
| 2 | 248 | 8 | 4 | 5 | NA | NA |
| 2 | 249 | 8 | 4 | 0 | NA | NA |
| 3 | 304 | 10 | 6 | 4 | 10 | 8 |
| 3 | 308 | 10 | 9 | 0 | 9 | 9 |
| 3 | 311 | 10 | 6 | 0 | 8 | 7 |
| 3 | 312 | 4 | 4 | 2 | 2 | 1 |
| 3 | 313 | 5 | 4 | 0 | 10 | 8 |
| 3 | 323 | 6 | 5 | 0 | 4 | 4 |
| 3 | 324 | 9 | 5 | 5 | 10 | 8 |
| 3 | 329 | 5 | 5 | 0 | 2 | 1 |
| 3 | 330 | 10 | 9 | 0 | 8 | 5 |
| 3 | 333 | 10 | 7 | 0 | 10 | 9 |
| 3 | 335 | 0 | 0 | 0 | 2 | 1 |
| 3 | 337 | 9 | 6 | 0 | 7 | 2 |
| 3 | 339 | 6 | 3 | 3 | 8 | 7 |
| 3 | 343 | 9 | 5 | 0 | 4 | 1 |
